# *In Vitro* Osteogenic Potential of Green Fluorescent Protein Labelled Human Embryonic Stem Cell-Derived Osteoprogenitors

**DOI:** 10.1155/2016/1659275

**Published:** 2016-11-28

**Authors:** Intekhab Islam, Gopu Sriram, Mingming Li, Yu Zou, Lulu Li, Harish K. Handral, Vinicus Rosa, Tong Cao

**Affiliations:** ^1^Oral and Maxillofacial Surgery, Faculty of Dentistry, National University of Singapore, Singapore; ^2^Oral Sciences, Faculty of Dentistry, National University of Singapore, Singapore; ^3^Experimental Dermatology Group, Institute of Medical Biology, Agency for Science, Technology and Research, Singapore

## Abstract

Cellular therapy using stem cells in bone regeneration has gained increasing interest. Various studies suggest the clinical utility of osteoprogenitors-like mesenchymal stem cells in bone regeneration. However, limited availability of mesenchymal stem cells and conflicting evidence on their therapeutic efficacy limit their clinical application. Human embryonic stem cells (hESCs) are potentially an unlimited source of healthy and functional osteoprogenitors (OPs) that could be utilized for bone regenerative applications. However, limited ability to track hESC-derived progenies* in vivo* greatly hinders translational studies. Hence, in this study, we aimed to establish hESC-derived OPs (hESC-OPs) expressing green fluorescent protein (GFP) and to investigate their osteogenic differentiation potential* in vitro*. We fluorescently labelled H9-hESCs using a plasmid vector encoding GFP. The GFP-expressing hESCs were differentiated into hESC-OPs. The hESC-OPs^GFP+^ stably expressed high levels of GFP, CD73, CD90, and CD105. They possessed osteogenic differentiation potential* in vitro* as demonstrated by increased expression of* COL1A1*,* RUNX2*,* OSTERIX*, and* OPG* transcripts and mineralized nodules positive for Alizarin Red and immunocytochemical expression of osteocalcin, alkaline phosphatase, and collagen-I. In conclusion, we have demonstrated that fluorescently labelled hESC-OPs can maintain their GFP expression for the long term and their potential for osteogenic differentiation* in vitro*. In future, these fluorescently labelled hESC-OPs could be used for noninvasive assessment of bone regeneration, safety, and therapeutic efficacy.

## 1. Introduction

Treatment of posttraumatic or neoplastic bone defects is a major reconstructive challenge. Recent developments in cellular therapy aim to mimic the process of bone repair by delivering cells capable of differentiating into osteoblasts [1]. Sources of such osteoprogenitors in the body include the bone marrow stromal cells (bMSCs) and extraskeletal sources such as adipose-derived stem cells and dental pulp stem cells [2]. Although evidence suggests that bMSCs are the primary source of osteoprogenitor cells [3], their acquisition is complicated by an invasive procedure and inadequate viable cell recovery due to an age-related decline in the number of osteoprogenitor cells [4]. Furthermore, the use of these stem cell sources is limited due to donor availability, healing complications at the donor site, and loss of proliferation capacity upon* in vitro* expansion [5].

Human embryonic stem cells (hESCs) have recently been proposed as attractive candidate for cellular therapy [6, 7]. In addition, to its hope for cellular therapies, hESCs also provide a potential* in vitro *human model to understand early human development and model diseased states and a potential tool for drug screening and toxicology studies [8]. hESCs possess a normal karyotype and maintain high telomerase activity resulting in its indefinite self-renewal potential and possibility for virtually unlimited expansion* in vitro* [9]. Further, hESCs can differentiate into cell types of all three germ layers [9, 10]. Hence, hESCs could be utilized to as potentially unlimited source of healthy and functional osteoprogenitors [10]. However, the clinical utility of these stem cells are limited by safety and ethical concerns [6, 7]. Secondly, the limited ability to track the hESC-derived cellular progenies* in vivo* greatly hinders translational studies. Though evidences suggest the clinical utility of osteoprogenitors-like mesenchymal stem cells in bone regeneration, the exact mechanism is poorly understood. It is proposed that osteoprogenitors could play a role in bone regeneration through homing to the site of injury, cell-cell interactions, and secretion of soluble paracrine factors. Furthermore, lack of reliable markers* in vivo* restricts the tracking of these cells after transplantation.

Methods such as flow cytometry, immunocytochemistry, and histology have been reliable in characterizing osteogenic differentiation* in vitro* and* in vivo* [11]. However, their application is restricted, as they do not allow online monitoring. Fluorescent tags like green fluorescent protein (GFP) allow visualization and real-time monitoring without the need for cellular fixation and immunostaining. Further, they are also valuable tools to control stem cell fate and study cell behaviour during differentiation. Hence, various transgenic hESC lines with constitutive or inducible tissue, cell-specific, or gene-specific fluorescent reporters have been generated by various groups [12–16]. Recently, lentiviral-based transduction and targeted gene knock-in methods, like zinc finger nucleases (ZFNs), transcription activator-like effector nucleases (TALENs), and clustered regulatory interspaced short palindromic repeats system (CRISPR/CAS9), are increasingly used as effective tools for gene delivery to hESCs. These methods offer several advantages including high transfection rates, capability for stable transgene expression and/or ability to generate cell-specific promoter-based reporter systems, and targeted genome editing [14–17]. However, some of the major concerns with these systems include insertional mutagenesis, viral integrations, and other off-target effects that can cause genomic instability and disruption of normal gene function [18]. In this regard, nonviral plasmid-based gene delivery methods provide an alternative platform. Nevertheless, in the context of hESCs, nonviral systems are associated with low transfection efficiency which is to certain extent overcome by single cell dissociation of hESCs and small molecule-based methods [19].

In the current study, we assess the ability of fluorescently labelled hESCs (using single cell dissociation and small molecule-mediated nonviral plasmid-based system) to differentiate into osteoprogenitors (hESC-OPs) and investigate their osteogenic differentiation potential.

## 2. Materials and Methods

### 2.1. Culture of hESCs

#### 2.1.1. Feeder-Dependent Culture of hESCs

The NIH-registered H9-hESC cell line, isolated and established at the University of Wisconsin, was used in this study. These cells were cultured on feeder-dependent system using mitomycin-C inactivated murine embryonic fibroblasts (MEFs) and hESC medium as described previously [20]. Briefly, the hESC medium consisted of Dulbecco's modified Eagle's medium (DMEM)/Ham's F12 (1 : 1) supplemented with 20% Knockout Serum Replacement (KO-SR; GIBCO), 1% (vol/vol) nonessential amino acids (Sigma-Aldrich), 1 mM L-glutamine (GIBCO), 4 ng/mL basic fibroblast growth factor (bFGF; Invitrogen), and 0.1 mM *β*-mercaptoethanol (Sigma-Aldrich). Media were changed every other day and passaged every 6-7 days using 1 mg/mL collagenase type IV (GIBCO) for 5 minutes, followed by manual dissociation to small clumps and seeding onto MEF-seeded plates.

#### 2.1.2. Fluorescent Labelling of hESCs

H9-hESC colonies were dissociated into single cells using 5-minute incubation with Accutase (StemCell Technologies), suspended in hESC medium supplemented with Y-27632 (inhibitor of Rho-associated protein kinase, ROCK, 10 *μ*M, Stemgent), and seeded onto MEF-seeded six-well plates. Plasmid construct consisted of pAcGFP1-1 backbone with pCAG-GFP promoter (Clonetech). hESCs were transfected with GFP plasmid using X-tremeGENE HP lipid-based transfection reagent (Roche) according to manufacturer's instructions. Briefly, 2 *μ*g of plasmid DNA and 6 *μ*L of X-tremeGENE HP transfection reagent in 200 *μ*L of hESC medium were incubated for 15 minutes at room temperature and added drop-by-drop to freshly seeded hESCs. Cells were cultured normally with daily change of media. 72 hours after transfection, GFP positive hESCs were selectively picked and transferred to new wells for expansion. Selection process was repeated until relatively homogenous GFP positive hESCs colonies were established over the next 5 passages. These hESCs stably expressed GFP for over 30 passages and are referred to as H9-hESC^GFP+^.

#### 2.1.3. Feeder-Free Culture of Fluorescently Labelled hESCs

To establish a feeder-free system, H9-hESCs^GFP+^ were transitioned and subcultured over Matrigel™ (BD Biosciences) in complete mTeSR™1 medium (StemCell Technologies). Under these conditions, the hESCs maintained their undifferentiated state and also stably expressed GFP. Confluent feeder-free hESC cultures were passaged every 5-6 days using 1 mg/mL Dispase (StemCell Technologies) for 5 minutes, followed by manual dissociation to small clumps and replating onto freshly prepared Matrigel coated plates as previously described [21].

### 2.2. Differentiation of Fluorescently Labelled hESCs to hESC-OPs

#### 2.2.1. Phase 1: Spin-EB Formation Using Forced Aggregation

H9-hESC^GFP+^ colonies were dissociated into single cells using 10–15-minute incubation with Accutase. Predetermined numbers of single cell dissociated hESCs (6 × 10^6^ cells) were seeded onto each well of Aggrewell™ 800 plates (StemCell Technologies) in Aggrewell media (StemCell Technologies) supplemented with Y27632 (10 *μ*M). The single cell dissociated cells were forcibly aggregated onto the microwells by centrifugation at 100 ×g for 3 minutes. The single cell dissociated hESCs formed clusters of uniform size within the microwells after overnight incubation. These clusters of hESCs would be referred to as spin-embryoid bodies (spin-EBs). The spin-EBs were harvested after 24 hours and transferred to ultralow-attachment six-well culture plates (Corning) in Aggrewell media for 5 days.

#### 2.2.2. Phase 2: Differentiation of Spin-EBs into Osteoprogenitors

After 5 days of differentiation, spin-EBs were collected and plated onto 1 *μ*g/cm^2^ fibronectin (GIBCO) coated tissue culture plates in Aggrewell medium until confluence (approximately 15–20 days). After confluence, the spin-EB outgrowths were trypsinized and cultured in Mesenchymal Stem Cell Growth Medium (PromoCell) for 3–8 passages. The EB outgrowths attained homogenous population of spindle-shaped cells from 3rd passage. These spindle-shaped cells were characterized for osteoprogenitor-related markers and would be referred to as H9-hESCs^GFP+^ derived osteoprogenitors (hESC-OPs^GFP+^).

### 2.3. RNA Extraction and Real-Time Reverse Transcriptase-Polymerase Chain Reaction (qRT-PCR)

Total RNA was extracted using RNeasy Plus Mini Kit (Qiagen) according to the manufacturer's protocol. Then, 500 ng of total RNA was used to generate cDNA using iScript cDNA synthesis kit (BioRad) and the cDNA was used as template for qRT-PCR. Relative expression levels of respective genes were analyzed using StepOne Plus™ real-time thermocycler (Applied Biosystems) and Fast SYBR Green PCR Master Mix System (Applied Biosystems). For relative quantification, the expression levels of respective genes were normalized to that of *β*-Actin and expressed as a fold change relative to the expression levels in undifferentiated hESCs.

### 2.4. Flow Cytometry Analysis

The differentiated hESCs (hESC-OPs^GFP+^) were dissociated into single cells using Accutase (StemCell Technologies) for 5 minutes at 37°C, resuspended in flow cytometry buffer (PBS containing 0.5% bovine serum albumin), and passed through 40 *μ*m cell strainer. Nonspecific binding of the antibodies was inhibited by incubating the cells with FcR*γ* blocking agent (Miltenyi Biotec) for 10 mins at 4°C. For labelling of cell surface antigens, the cells were incubated with antibodies against CD73-APC (eBioscience), CD90-PE (BD Pharmingen), CD105-PE (BD Pharmingen), and CD45-PerCP (BD Pharmingen) at 4°C for 10 minutes. After washing with flow cytometry buffer, the antibody-labelled cells were analyzed using DakoCytomation Cyan ADP and Summit v4.3 software.

### 2.5. Osteogenic Differentiation of hESC-OPs^GFP+^

hESC-OPs^GFP+^ were differentiated towards osteogenic lineage using MSC osteogenic differentiation medium (PromoCell). Briefly, hESC-OPs^GFP+^ were plated in a tissue culture plates (seeding density: 3 × 10^4^ cells/cm^2^) using MSC growth medium. When the cells were almost 100% confluent (1-2 days), osteogenic differentiation was induced with MSC osteogenic differentiation medium (PromoCell) as per manufacturer's instructions. MSC growth medium was used as a negative control. The cells were incubated for 14–28 days, with media changed every third day.

### 2.6. Alizarin Red Staining

Alizarin Red staining was used to identify calcific nodules produced by cells after 28 days of osteogenic differentiation. Cultures were washed with PBS and fixed with 4% paraformaldehyde for 20 minutes at room temperature. Cultures were then washed and incubated with Alizarin Red for one minute, washed thoroughly, and air-dried. Formation of chromogenic complex between o-cresolphthalein and calcium ions was visualized using inverted light microscope (Olympus IX70).

### 2.7. Immunocytochemical Staining

hESC-OPs^GFP+^ cells differentiated under osteogenic conditions for 14 days were washed with PBS and fixed in 4% paraformaldehyde for 20 minutes at room temperature. For detection of intracellular proteins, the cells were permeabilized with 0.1% Triton X-100 (Sigma-Aldrich) in PBS and blocked with 10% goat serum/2% BSA in PBS for 1 hour at room temperature. Then the cells were incubated with primary antibodies against mouse anti-human GFP (1 : 200, Santa Cruz Biotech), rabbit anti-human collagen-1 (1 : 300, Millipore), rabbit anti-human osteocalcin (1 : 200, AbD Serotec), and rabbit anti-human alkaline phosphatase (1 : 200, Santa Cruz Biotech) in blocking solution at 4°C overnight. Then, the cultures were washed thrice in PBST (0.1% Tween-20 in PBS) and incubated with goat anti-mouse Alexa Fluor-488 and goat anti-rabbit Alexa Flour-594 conjugated secondary antibodies (1 : 200, Molecular Probes) for 1 hour at room temperature, followed by nuclear labelling with 4′,6-diamidino-2-phenylindole dihydrochloride (DAPI) for 5 minutes at room temperature. The cultures were then washed thrice with PBS. Immunostained cultures were observed and imaged using Olympus IX70 fluorescence microscope.

### 2.8. Statistical Analysis

The results of qRT-PCR are presented as mean ± standard deviation of three experiments. Statistical differences were evaluated by a two-tailed Student's *t*-test. *p* values <0.05 were considered as statistically significant.

## 3. Results

### 3.1. Differentiation of Fluorescently Labelled hESCs into hESC-OPs

**Figure 1:**
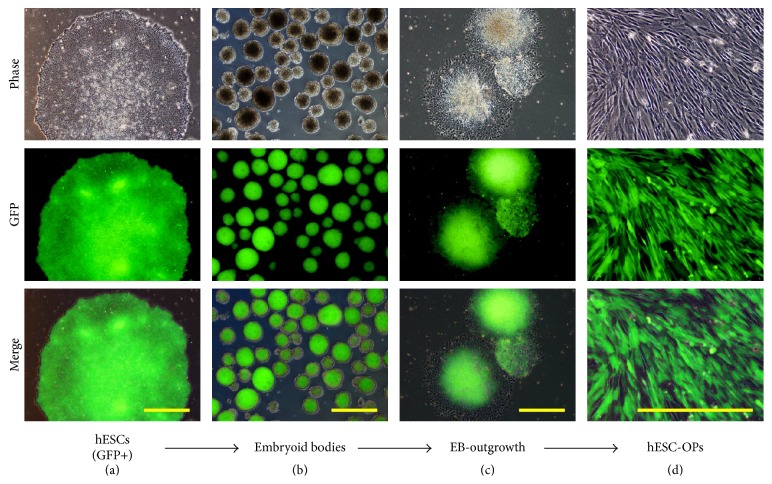
Differentiation of GFP-expressing hESCs into hESC-OPs. The phase contrast, fluorescent, and merged photomicrographs demonstrate the differentiation of GFP-expressing hESCs (a) into embryoid bodies (b), embryoid body outgrowth, (c) and hESC-OPs (d). Scale bar: 500 *μ*m.

To generate fluorescently labelled osteoprogenitor-like cells from hESCs, H9-hESCs were first fluorescently labelled with GFP and adapted to feeder-free culture system (Figure 1(a)). These H9-hESCs^GFP+^ were differentiated through a two-stage EB-outgrowth method under serum-free conditions similar to a method previously described for differentiation of hESCs to MSC-like phenotype [10, 22]. Briefly, spin-EBs were generated by forced aggregation of single cell dissociated hESC colonies followed by suspension culture of hESC aggregates under ultralow-attachment and serum-free conditions (Figure 1(b)). After 5 days of differentiation under suspension culture, spin-EBs were plated onto fibronectin-coated plates for outgrowth of differentiated cells (Figure 1(c)). Immediately after adherent culture of spin-EBs, cells with different morphologies varying from epithelioid to spindle-shape migrated out from the spin-EBs. Upon serial subculture, spindle-shaped cells outnumbered other cell types and attained homogeneous morphology after 3 passages (Figure 1(d)). The hESC-derived cells stably expressed GFP throughout the differentiation process resulting in the differentiation of fluorescently labelled hESCs (H9-hESCs^GFP+^) to osteoprogenitor-like cells (hESC-OPs^GFP+^) (Figure 1).

### 3.2. Characterization of Fluorescently Labelled hESC-OPs

**Figure 2:**
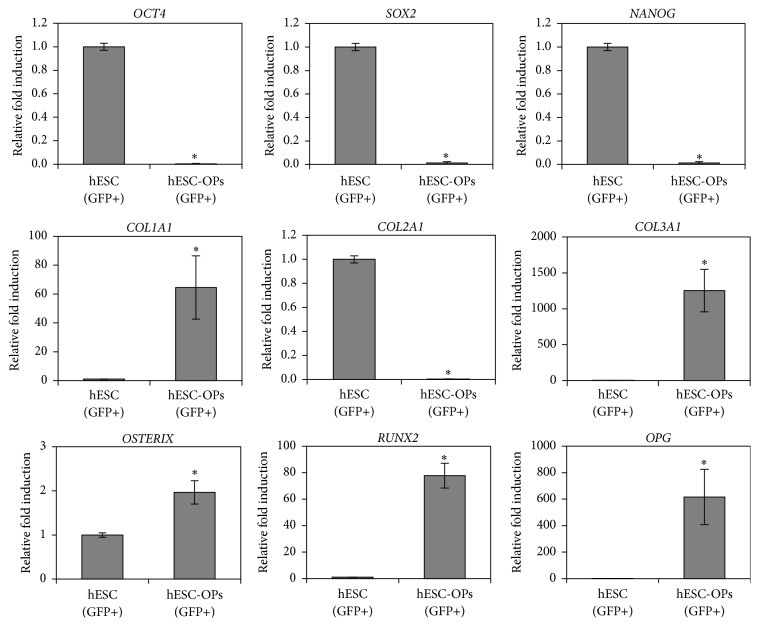
Characterization of hESC-OPs (GFP+) by real-time RT-PCR for pluripotency (*OCT4, SOX2,* and* NANOG*), mesenchymal (*COL1A1 *and* COL3A1*), chondrogenic (*COL2A1*), and osteogenic (*RUNX2*,* OSTERIX*, and* OPG*) lineage-associated transcripts. The transcript levels were normalized to respective *β*-*Actin* levels and to hESC (GFP+) cells. Values represent the means ± SD of three experiments (^*∗*^ *p* < 0.05 versus hESC control).

The phenotype of fluorescently labelled hESC-OPs^GFP+^ was characterized using qRT-PCR and flow cytometry. qRT-PCR analysis of hESC-OPs^GFP+^ demonstrates the downregulation of transcripts related to pluripotency (*OCT4*,* SOX2*, and* NANOG*) and chondrogenic (*COL2A1*) lineage accompanied by upregulation of mesenchymal (*COL1A1* and* COL3A1*) and osteogenic (*OSTERIX*,* RUNX2*, and* OPG*) lineage-associated transcripts (Figure 2).

**Figure 3:**
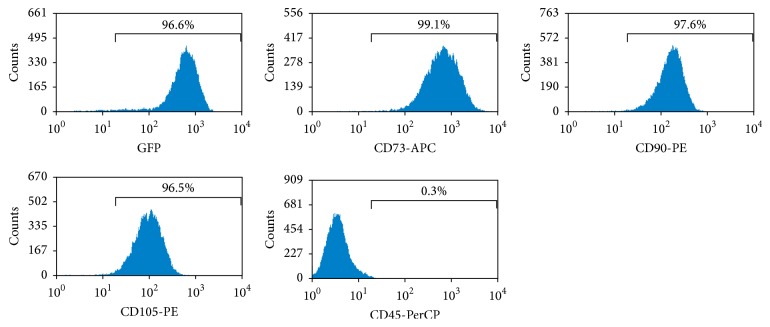
Immunocharacterization of hESC-OPs (GFP+) using flow cytometry for expression of surface markers.

The immunophenotype of hESC-OPs^GFP+^ was analyzed using flow cytometry for surface markers. The hESC-OPs^GFP+^ demonstrated strong expression of GFP (96.6%) and mesenchymal stem cell-associated surface markers CD73 (99.1%), CD90 (97.6%), and CD105 (96.5%). These cells were negative for hematopoietic marker CD45 (<1%) (Figure 3).

### 3.3. Characterization of Osteogenic Differentiation of Fluorescently Labelled hESC-OPs

**Figure 4:**
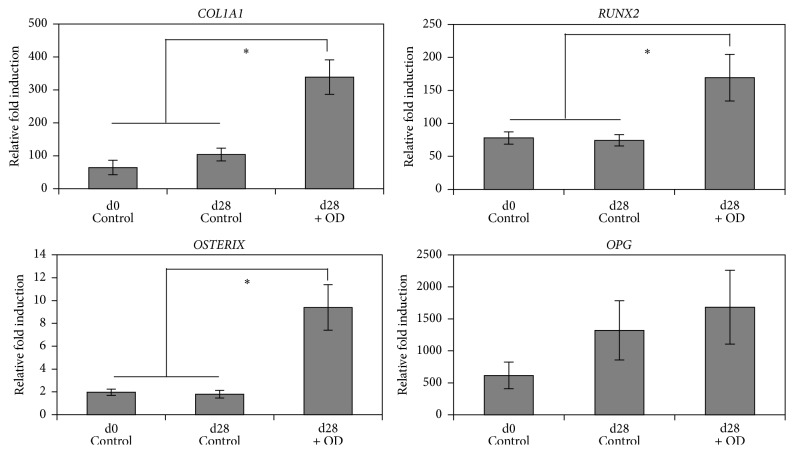
Characterization of osteogenic differentiation of hESC-OPs (GFP+) by real-time RT-PCR. The transcript levels were normalized to respective *β*-*Actin* levels and to hESC (GFP+) cells. Values represent the means ± SD of three experiments (^*∗*^ *p* < 0.05 versus respective controls). hESC-OPs (GFP+) were differentiated for 4 weeks in the presence of osteogenic factors (OD). Day 0 hESC-OPs (GFP+) and cells differentiated for 4 weeks without osteogenic factors were used as controls.

**Figure 5:**
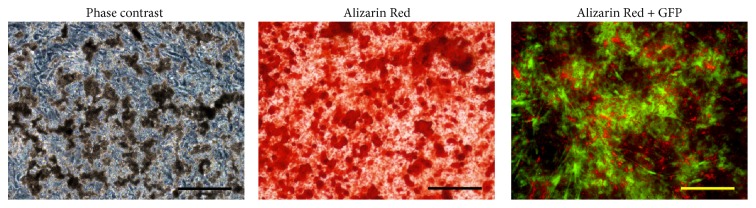
Osteogenic differentiation of GFP-expressing hESC-OPs demonstrated using Alizarin Red staining. Scale bar: 200 *μ*m.

**Figure 6:**
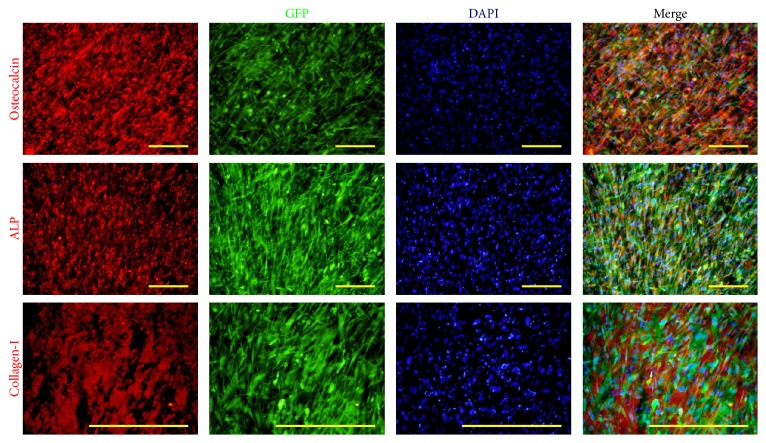
Osteogenic differentiation of GFP-expressing hESC-OPs demonstrated using immunocytochemical staining for osteocalcin, alkaline phosphatase (ALP), and collagen-I after 14 days of induction in osteogenic differentiation conditions. Scale bar: 500 *μ*m.

The osteogenic differentiation ability of hESC-OPs^GFP+^ was characterized using qRT-PCR, Alizarin Red staining, and immunocytochemistry. qRT-PCR analysis demonstrates the significant upregulation of transcripts (*COL1A1*,* RUNX2*, and* OSTERIX*) associated with osteogenic differentiation except OPG (Figure 4). Under osteogenic conditions, hESC-OPs^GFP+^ formed large mineralized nodules that stained intensely red with Alizarin Red (Figure 5). Immunocytochemical staining of hESC-OPs^GFP+^ differentiated under osteogenic conditions for 14 days demonstrates the strong expression of osteocalcin, alkaline phosphatase, and collagen-I (Figure 6).

Overall, these results suggest that the efficient differentiation of fluorescently labelled hESCs into hESC-OPs stably expressed GFP and demonstrated osteogenic differentiation* in vitro*.

## 4. Discussion

Poor survival of transplanted cells and safety concerns related to formation of ectopic tissues and tumors raise the crucial need for establishing an effective technique for tracing the hESCs and their differentiated progenies* in vivo* for tissue engineering related applications. Fluorescent dyes, such as DiL, Hoechst 33342, CFSE (carboxyfluorescein succinimidyl ester), and PKH linker dyes, have been used to trace the presence and activity of transplanted cells [23–25]. However, these cytoplasmic or nuclear markers lose their intensity as the cells proliferate. In addition, these dyes could be taken up nonspecifically by the host cells* in vivo* and hence impair long-term tracing studies and may provide misleading information on donor survival and differentiation [23, 26].

The incorporation of GFP into the genome of hESCs that is stably expressed in the differentiated progenies provides a proof-of-concept to trace their presence, migration, and formation of tissues after transplantation [27]. An elegant study proved the existence of bipotent hemangioblasts that are capable of differentiating into endothelial and hematopoietic cell lineages using GFP-labelled hESCs-derived blast colonies transplanted into various mouse ischemia models [28]. Similarly, other studies have shown the utilization of fluorescently labelled hESC-derived endothelial cells to trace and evaluate the formation and integration of functional blood vessels* in vivo* [29, 30] and their transition to hematopoietic cells [31].

Due to the genomic integration of GFP, it is important to ensure the continued proliferation and differentiation ability of the stem cell population. Previous studies have shown successful transduction using lentiviral vectors encoding fluorescent proteins in human adipose-derived stem cells with potential for adipogenic and osteogenic differentiation [32] and in human placenta-derived MSCs with potential for adipogenic, osteogenic, and hepatic differentiation [33]. In a recent study, Ovchinnikov et al. used a lentiviral-based delivery system to generated transgenic pluripotent stem cells (PSC) lines that expressed pluripotency-driven GFP [13]. These cell lines effectively report for pluripotency and hence play a role in detection of PSCs from their differentiated progenies. Similarly, Zou et al. generated a RUNX2 promoter driven YFP based reporter system in hESCs to track their commitment to osteogenic lineage [15]. Using these transgenic hESCs, they elegantly demonstrated that BMP2 does not induce osteogenic differentiation but promotes the generation of CD73+ osteoprogenitors and their osteogenic differentiation. In a recent study, Zhou et al. generated choline acetyl transferase (ChAT) promoter driven zsGreen expressing hESC line that labels cholinergic neuronal differentiation [14]. Overall, these reporter hESC lines not only provide an opportunity to monitor lineage specific differentiation but also aid in study of physiological and biochemical pathways controlling the differentiation. In this study, we have shown the stable expression of GFP in hESCs and their differentiated progeny (hESC-OPs). The expression of surface markers (CD73, CD90, and CD105), data from gene expression analysis, and Alizarin Red and immunofluorescent staining demonstrate the osteogenic commitment and differentiation potential of these GFP-expressing hESCs. These results are in agreement with previous studies [10, 15, 20, 34, 35]. These data also suggest the stable expression of GFP in hESC-OPs and the genetic manipulation did not affect their osteogenic differentiation.

An elegant study demonstrated the comparative efficiency and effect of different viral and nonviral vectors on rat MSCs and their differentiation potential [36]. This study demonstrated superior efficacy of gene transfer and lower toxicity using lentiviral-based methods compared to adenoviral based methods. Further, transfection using plasmid DNA is moderately effective compared to the high efficiency of transduction using lentiviral-based methods of gene transfer. Similarly, another study using human MSCs has demonstrated the higher efficiency of lentiviral-based transduction of GFP compared to oncoretroviral vectors [37]. However, the lentiviral GFP transduction seemed to impair* in vitro *osteogenesis to a certain extent, which might be due to lentiviral factors or GFP gene itself. It is also known that GFP can be toxic to certain cell types resulting in lack of protein expression [38] and phenotypic changes [39] or have subtle effects on differentiation [37]. Conventionally, nonviral plasmid DNA vectors are less efficient in gene transfers but are advantageous due to their lower toxicity. In the context of hESCs, this is important as hESCs upon dissociation into single cells are extremely prone for cell death and apoptosis. Recently, Yen et al. developed a nonviral, plasmid-based fluorescently labelling of hESCs with moderately high transfection efficiencies through single cell dissociation of hESCs and use of small molecules during the exposure to plasmid [19]. In this study, we used a similar approach to transfect the hESCs. However, the efficiency of the transfection were not quantified as the selection process was carried out through manual selection over several passages.

The ability of fluorescently labelled hESCs to differentiate to hESC-OPs and further to osteogenic lineage provides a platform to trace the osteogenic potential in tissue engineering related applications. However, additional studies are necessary to determine the engraftment and* in vivo *bone regeneration potential of these fluorescently labelled hESC-OPs. However, we do not propose to utilize the fluorescently labelled hESC progenies for human clinical translational applications. The use of GFP-labelled hESC-OPs would be extremely useful in noninvasive imaging of bone regeneration within three-dimensional (3D)* in vitro* scaffolds and* in vivo *animal models. Recently, several studies have produced bone-like matrix by seeding human PSCs on different 3D scaffolds [40–43] and using perfusion bioreactors [44, 45]. Jeon et al. used a biomimetic approach to regenerate bone by 3D coculture of human PSC-derived osteoblasts and osteoclasts within a synthetic scaffolds [46]. We have recently developed protocols to direct the differentiation of hESCs to vascular cells [20, 47]. In future studies, we plan to address the osteodifferentiation potential of hESC-OPs cocultured with vascular cells within 3D scaffolds to generate vascularized bone tissues.

## 5. Conclusion

In this study, we have demonstrated that hESCs fluorescently labelled using plasmid vectors encoding GFP can be efficiently differentiated into hESC-OPs. These hESC-OPs maintain their GFP expression for the long term and their potential for osteogenic differentiation* in vitro*. Plasmid-based GFP transfection provides a direct and simple method to label hESCs and their progenies and does not impede their* in vitro* differentiation potential. This technique provides opportunities to investigate bone regeneration using noninvasive imaging modalities. However, extensive long-term* in vivo *studies are required to access the safety, efficacy, and therapeutic potential of these cells.
